# The Effect of Benzyl Isothiocyanate on the Expression of Genes Encoding NADH Oxidase and Fibronectin-Binding Protein in Oral Streptococcal Biofilms

**DOI:** 10.3389/froh.2022.863723

**Published:** 2022-04-11

**Authors:** Hawraa Alhandal, Esraa Almesaileikh, Radhika G. Bhardwaj, Areej Al Khabbaz, Maribasappa Karched

**Affiliations:** ^1^Oral Microbiology Research Laboratory, Department of Bioclinical Sciences, Faculty of Dentistry, Health Sciences Center, Kuwait University, Kuwait, Kuwait; ^2^Department of Surgical Sciences, Faculty of Dentistry, Health Sciences Center, Kuwait University, Kuwait, Kuwait

**Keywords:** biofilm, Miswak, antibacterial agents, oral health, gram-positive bacteria, gene expression, streptococci

## Abstract

Recent studies have shown that antimicrobial treatment results in up- or down regulation of several virulence-associated genes in bacterial biofilms. The genes encoding NADH oxidase (*nox*) and fibronectin-binding protein (*fbp*) are known to play important roles in biofilm growth of some oral bacterial species. The objective was to study the effect of benzyl isothiocyanate (BITC), an antimicrobial agent from Miswak plant, on the expression of *nox* and *fbp* genes in some oral streptococci. The biofilms were treated with BITC and mRNA expression of *nox* and *fbp* genes was measured by comparative ΔΔCt method. The highest amount of biofilm mass was produced by *A. defectiva*, followed by *S. gordonii, S. mutans, G. elegans* and *G. adiacens*. Upon treatment with BITC, *S. gordonii* biofilms showed highest folds change in mRNA expression for both *fbp* and *nox* genes followed by *S. mutans, A. defectiva*, and *G. adiacens*. *G. elegans* mRNA levels for *nox* were extremely low. In conclusion, BITC treatment of the biofilms caused an upregulation of biofilm-associated genes *fbp* and *nox* genes in most of the tested species suggesting the significance of *these* genes in biofilm lifestyle of these oral bacteria and needs further investigation to understand if it contributes to antimicrobial resistance.

## Introduction

Biofilms are a complex and functional community of microorganisms encased in a polymeric matrix and attached to one another or to a solid surface. A characteristic feature of microbial biofilms is the secretion of an adhesive matrix of highly hydrated extracellular polymeric substances (EPSs), comprised of polysaccharides, proteins, lipids, and nucleic acids [1]. Biofilms are structurally and physiologically complex entities. Within the biofilms, microorganisms coordinate and cooperate like multicellular organisms [2]. Ability of bacterial species to form biofilm is regarded a key virulence factor. Biofilm-associated bacterial infections in medicine have been extensively studied [3, 4].

Dental plaque develops naturally, but it is also associated with two of the most prevalent diseases affecting the people worldwide, which are caries and periodontal diseases [5]. Dental plaque is the diverse microbial community that develops on the tooth surface embedded in a matrix of polymers of bacterial and salivary origin [6]. Plaque formation starts once a tooth surface is cleaned and the early colonizers adhere to the acquired enamel pellicle, which is a thin layer of salivary glycoproteins deposited on tooth surface. Subsequently, secondary colonizers attach to the early colonizers through specific molecular interactions known as co-aggregation [6, 7]. These can involve protein-protein or carbohydrate-protein interactions, and this process determines the pattern of bacterial succession.

The oral cavity contains complex, multispecies microbial communities [8, 9]. This indicates that the residents in this community should exhibit extensive interactions while forming biofilm structures, carrying out physiological functions, and inducing microbial pathogenesis. Interspecies interactions in biofilms are competitive, cooperative and antagonistic [10]. In addition, such interactions may have specific effects in terms of the virulence properties of biofilm residents, which could influence the overall pathogenicity of biofilms.

Even though it is known that biofilms generally are more resistant to antibiotics, they can affect bacterial biofilms at different levels, including changes in the overall structure of the population, selection of resistant organisms, or alterations in bacterial physiology. Recent studies have shown that antibiotic treatment results in up- or down regulation of several genes [11]. However, little is known how antimicrobials, which have a general and broader mechanism of antibacterial action, influence the expression of biofilm-associated genes [12, 13]. The genes encoding NADH oxidase (*nox*) [14] and fibronectin-binding protein (*fbp*) [15] are known to play important roles in biofilm formation of some oral bacterial species. In this study, we aim to study the effect of benzyl isothiocyanate [16], an antimicrobial compound from Miswak plant, on the expression of *nox* and *fbp* genes in oral streptococci.

## Methods

### Bacterial Strains and Culture Conditions

*Granulicatella elegans* CCUG 38949, *Granulicatella adiacens* CCUG 27809, and *Abiotrophia defectiva* CCUG 27639 were cultured on chocolate blood agar with 0.001% pyridoxal HCl [17] for 2 days at 37°C and 5% CO_2_. in air. While *Streptococcus mutans* CCUG 11877 and *Streptococcus gordonii* CCUG 33482 were cultured on brucella blood agar containing 5% sheep blood and incubated as above. Prior to harvesting the growth for each experiment, purity of cultures was checked by observing the plates under stereo microscope.

### *In silico*- and PCR-Confirmation of the Target Genes

Presence of the target genes encoding NADH oxidase and fibronectin binding protein was checked in the whole genome sequences of each species by using NCBI BLAST. Once confirmed, the two genes were PCR-amplified from each species. For PCR, primers were designed based on the obtained nucleotide sequences using the Bioinformatics Software Laser Gene Core Suite vs. 12 (Table 1). PCR reactions were performed on Veriti^TM^ PCR machine (Applied Biosystems) using Ready-To-Go PCR beads (GE Life Sciences) and reaction conditions mentioned as earlier [17]. Agarose gel electrophoresis of the PCR amplicons was performed as described [17] and the amplicons were finally visualized on a G:Box Imaging system (SynGene).

**Table 1:** Primers used in this study.

**Gene**	**Species**	**Sequence**	**Annealing temperature (°C)**	**Product size (bp)**	**References**
*fbp*	*G. elegans*	F: AGGCTTTTGCGATTGCTGCG	52	127	This study
		R: TGAGGAGTTGCGCCACGAAT			
	*G. adiacens*	F: AGGGGAGCTCTGGTTCCATGT	55	118	This study
		R: TGCTAATTCGGCAGCATCCGT			
	*A. defectiva*	F: GCCAGTCGACCCAGTCCTTG	55	137	This study
		R: CGGCCACCTCAAGATGAGCA			
	*S. mutans*	F: GTGGCGCCTCAATTAGTGGGA	55	106	This study
		R: GGTGTGCCGAACAGACAAGC			
	*S. gordonii*	F: ACCGATGAAACGATTGGGCA	55	102	This study
		R: CCGGTTTAGCTCCATTTGGC			
*nox*	*G. elegans*	F: GAACTTTGTCGCCCGCGATG	55	127	This study
		R: CAGGTGACTCCTGTGCGGTTA			
	*G. adiacens*	F: ACCGATGTATCCACCACCGA	55	109	This study
		R: TCCTCCAATTCCAGGACGTGA			
	*A. defectiva*	F: TGGTGGCGAATACGAAGCTGA	55	108	This study
		R: ACGATGATAGCACCGTTAGGCA			
	*S. mutans*	F: AACCAAGGAAAGAGATGTTTGA	50	127	[18]
		R: GGTGCTAACCACGCAGGTA			
	*S. gordonii*	F: GGGTTGTGGAATGGCACTTTGG	50	120	[19]
		R: CAATGGCTGTCACTGGCGATTC			
16S rRNA	All bacteria	F: GTGSTGCAYGGYTGTCGTCA	50, 55	147	[20]
		R: ACGTCRTCCMCACCTTCCTC			

### Biofilm Culture and Quantification

Biofilm cultures were set up as described previously [17]. Briefly, from the agar-grown cultures, colonies were harvested, suspended in brucella broth and the cell suspensions adjusted to OD_600_ = 1. A 100-μl aliquot from each bacterial cell suspension was added separately into wells of a 24-well cell culture plate containing 900-μl brucella broth. For *Granulicatella* and *Abiotrophia* species, 0.001% pyridoxal was added. Wells containing only broth was served as a negative control. Biofilm cultures were incubated for 3 days in 5% CO_2_ atmosphere at 37°C.

### Crystal Violet Staining of Biofilms

Following the incubation, broth supernatant was removed, and biofilms were washed twice gently with 1 ml sterile Phosphate Buffer Saline (PBS). One ml of crystal violet stain (2%) was applied to each well and incubated for 10 min. The stain was removed, and the wells were washed 6 times using PBS and airdried before taking pictures.

### Confocal Microscopy

After growing biofilms as described above, 1 ml of 4% paraformaldehyde solution (pH = 7.4) was added to each well and incubated for 30 min at room temperature for fixation. Supernatant was removed and the wells were washed twice with 1 ml of sterile PBS. To each well, 500-μl of a mixture containing 1.5 ml PBS and 4.5 μl Syto-9 dye was added. The plate was incubated in dark for 15 min and then the supernatant was removed and washed with PBS. The plate was dried, and a drop of mounting oil was added to each well and covered with 24 × 50 mm coverslip. Biofilms were visualized by confocal laser scanning microscopy (LSM 700, Carl-Zeiss).

### Effect of BITC on Biofilm Formation

The effect of BITC on the biofilm forming abilities of the bacterial species was studied by growing the biofilms in the presence of 10 μM BITC for 2 days under the same culture conditions as above. After incubation, 1 ml methanol was added to each well and the plate was kept undisturbed for 15 min at room temperature for fixing of the biofilms. Methanol was removed and the plate was air-dried for 45 min at room temperature. One ml of 0.1% aqueous crystal violet stain was added to each well and allowed to stay at room temperature for 20 min. Excess water was removed from the wells by tapping the plate upside down on a dry tissue paper. The plate was completely dried by keeping it open for 5–10 min in the fume hood. To each well, 500 μl 33% acetic acid was added and incubated at room temperature on a shaker for 5 min. One hundred microliter from each well was added into wells of a 96-well plate in triplicates and the absorbance was read at 590 nm using a microplate reader.

### Treatment of Biofilms With BITC

Biofilms of each bacterial species were grown as described above and benzyl isothiocyanate (Sigma) was added at a concentration of 10 μM [16] prepared in DMSO and allowed for 2 h at the same incubation conditions as above. For negative control, biofilms were treated with DMSO alone. Following the incubation, broth supernatants were removed, and the biofilms were scrapped off and collected into sterile micro centrifuge tubes. Immediately, RNA *later*® (~10 volumes of the approximate weight of biofilms) was added and all samples were stored at −80°C until used.

### RNA Isolation

For RNA isolation, the samples were taken out from the freezer and thawed on ice. After centrifuging at 5,000 × g for 5 min, the pellets were subjected to RNA isolation. Equal volume of RLT buffer (350 μl) and 70% ethanol (350 μl) were added and mixed gently with a pipet tip without vortexing. All the contents were transferred to mini spin columns and centrifuged at 8,000 × g for 15 s. The flow-through was discarded and the RNA bound to the membrane was washed with 700 μl wash buffers RW1. Five hundred microliter elution buffer RPE was added and centrifuged at 8,000 × g for 15 s and the flow-through was discarded. Another 500 μl elution buffer RPE was also added and centrifuged at 8,000 × g for 2 min. Finally, to elute the bounded RNA, 50 μl RNase-free water was added directly to the spin column membrane and centrifuged for 1 min at 8,000 × g. The concentration of RNA was determined by Nanodrop and the purity was assessed by A_260_/A_280_ ratio.

### Reverse Transcription Real-Time PCR

From the purified RNA stored at −20°C, cDNA was synthesized by using a High-Capacity cDNA Reverse Transcription Kit (ABI Systems) according to manufacturer's instructions. Expression levels of all target genes were normalized using the 16S rRNA gene. The reactions were performed on ABI 7500 Fast Real-Time PCR machine. The reaction mixture was as follows: 10 μl SYBR Green master mix (Power SYBR Green® Kit, Applied Biosystems), 1 μl each of forward and reverse primer (0.2 μM), 7 μl H_2_O and 1 μl DNA template. Temperature profile was as follows: a 10-min initial denaturation at 95°C followed by 40 cycles of 95°C for 15 s, 50–60°C (depending on the primer pair) for 30 s and 72°C for 30 s. Primers and conditions used in real-time PCR for mRNA gene expression analysis are mentioned in Table 1. Data from fluorescent signal was acquired at the elongation step and analyzed using the software SDS 2.3.0v. Analysis of the expression of *nox* and *fbp* genes was performed on cells from untreated control and biofilm treated groups by comparative ΔΔCt method in ABI 7500 SDS system (Applied Biosystems). The amount of target, normalized to endogenous (16S rRNA gene) and relative to calibrator (untreated), by 2^−ΔΔCt^ and the expression fold change was presented graphically.

### Statistics

All experiments were repeated 3 times. Biological and technical replicates were included in each experiment. Differences between groups were compared by Mann Whitney U test. A *P*-value <0.05 was regarded significant. SPSS vs25 for windows was used for statistical analysis.

## Results

### PCR Optimization for the Target Genes *nox* and *fbp*

**Figure 1:**
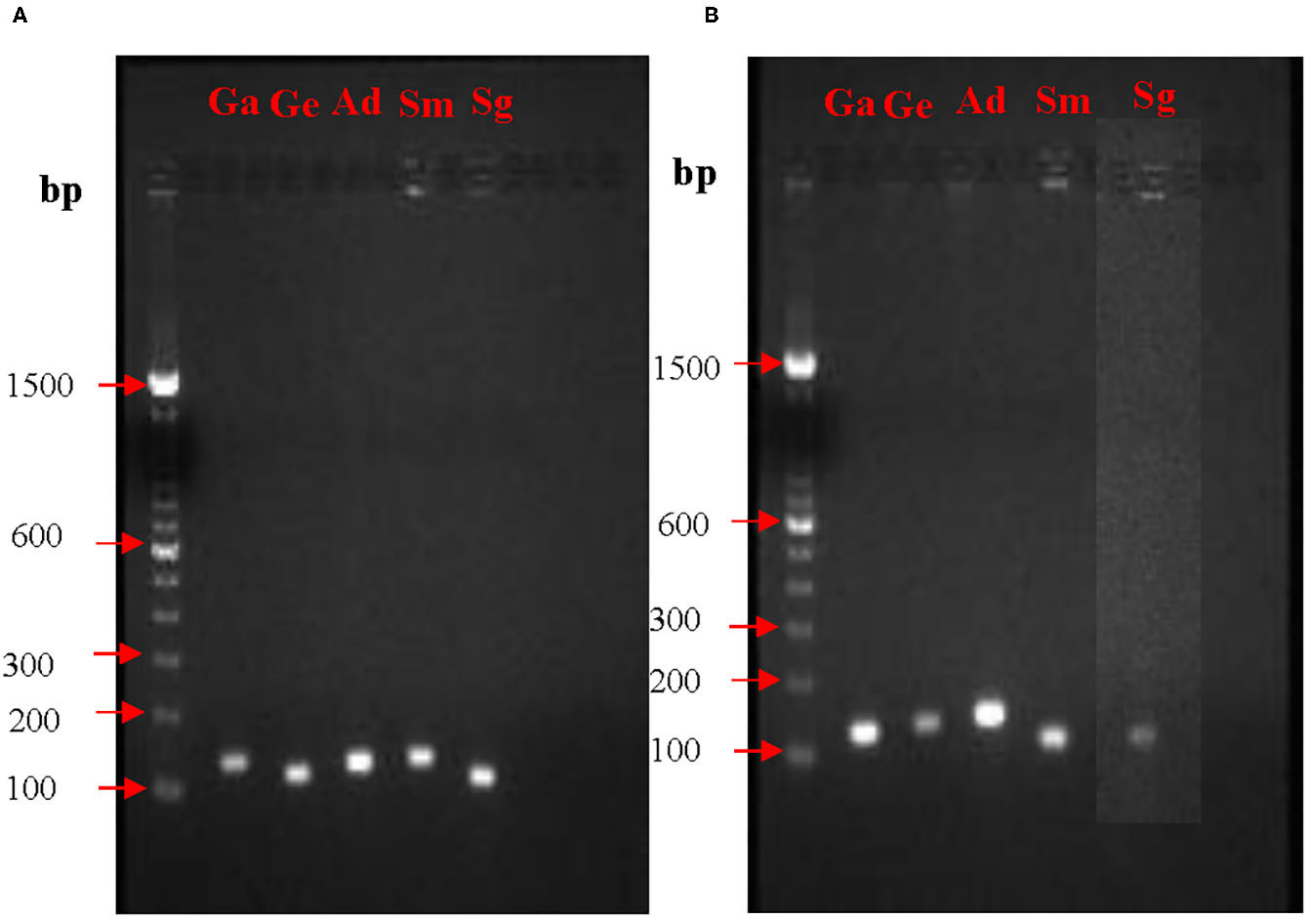
PCR optimization of *nox* **(A)** and *fbp* **(B)** genes. To confirm the amplification of *nox* and *fbp* genes from each strain, PCR and agarose gel electrophoresis were performed prior to RT-PCR experiments. bp, base pair; Ga, *G. adiacens*; Ge, *G. elegans*; Ad, *A. defectiva*; Sm, *S. mutans*; Sg, *S. gordonii*.

Prior to quantification of mRNA expression from *nox* and *fbp* genes, PCR conditions and the amplicon sizes were standardized. PCR products when analyzed on agarose gel electrophoresis showed single bands for all the five species. Expected sizes of the amplicons for the *fbp* gene visualized on the agarose gels were for *G. elegans* 127 bp, *G. adiacens* 118 bp, *A. defectiva* 137 bp, *S. mutans* 106 bp, and *S. gordonii* 102 bp. Similarly, expected sizes of the amplicons for the *nox* gene were for *G. elegans* 127 bp, *G. adiacens* 109 bp, *A. defectiva* 108 bp, *S. mutans* 127 bp, and *S. gordonii* 120 bp (Table 1). The absence of non-specific amplifications in all the tested species was evident from the single bands (Figure 1).

### Biofilm Visualization and the Effect of BITC on Biofilm Formation Ability of the Bacterial Species

**Figure 2:**
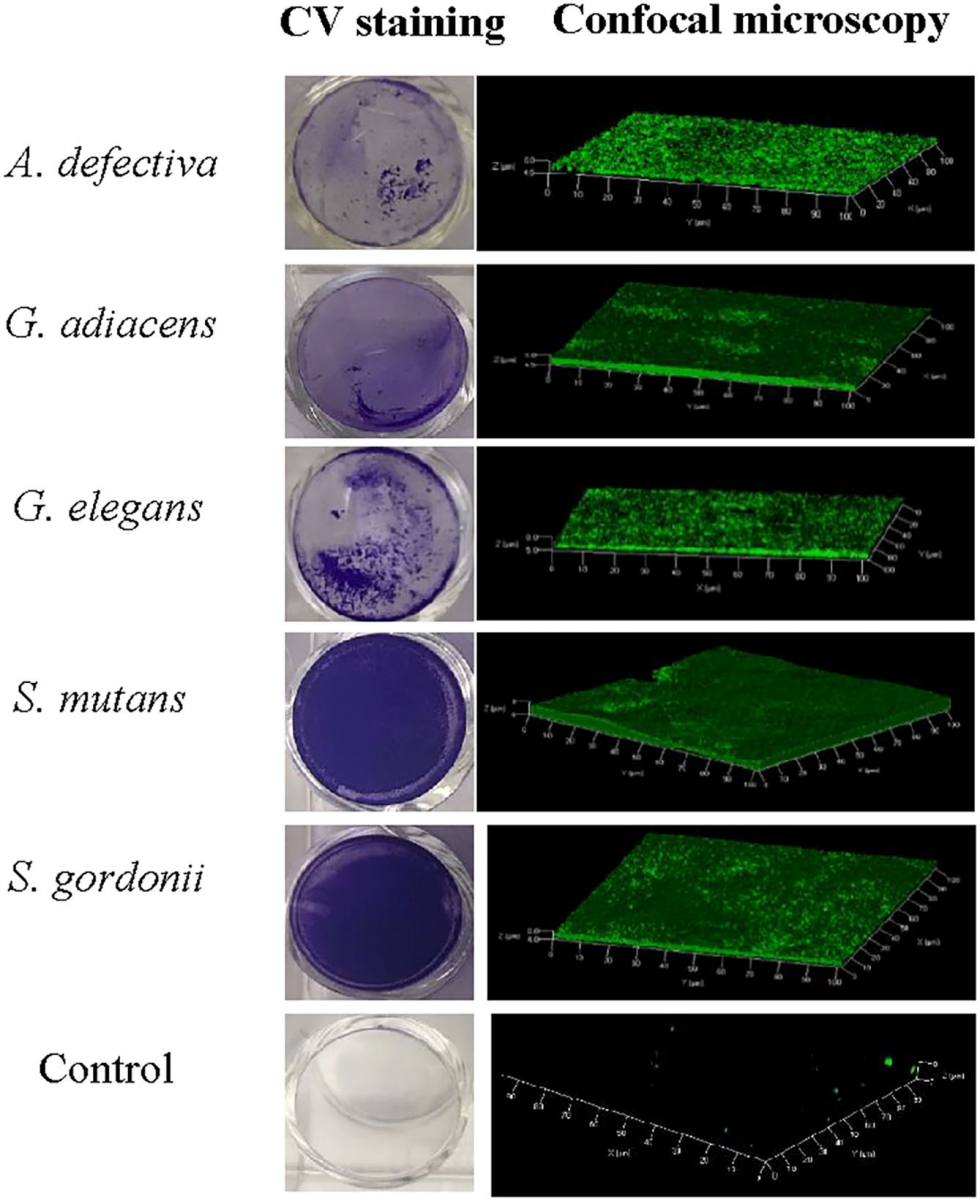
Confocal microscopy and crystal violet staining of monospecies biofilms of five oral streptococcal species. For confocal microscopy, bacteria were cultured in brucella broth in the wells of Millicell® EZ slides (Millipore) and stained with Syto9®. Images were acquired on Carl-Zeiss LSM 700 at 630× magnification and 3D view reconstructed using the software ZEN 2012. The bacteria were grown similarly for crystal violet staining except that 24-well cell culture plates (BD Bioscience) were used.

**Figure 3:**
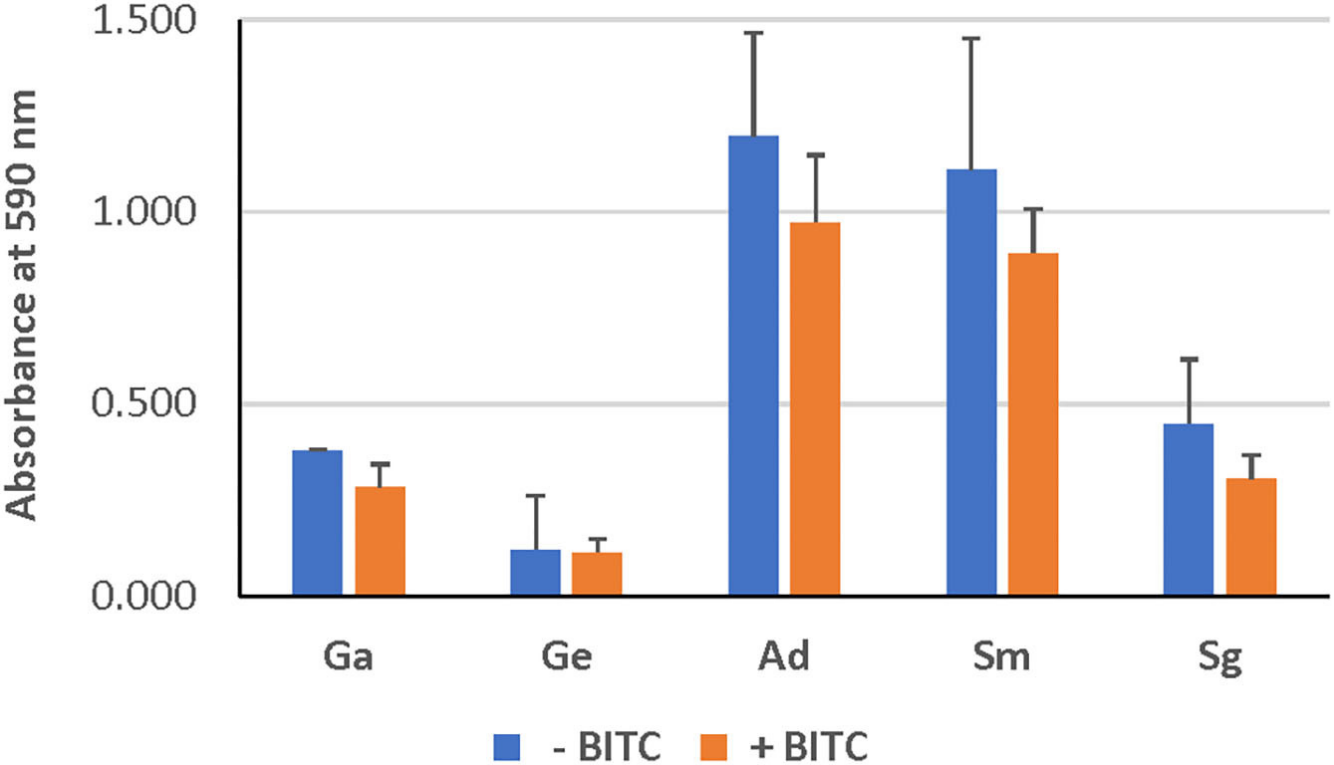
Effect of BITC treatment on the biofilms. The biofilm biomass was quantified by crystal violet staining after treating with BITC for 2 h in 5% CO_2_ at 37°C. The absorbance was measured at 590 nm wavelength. Ga, *G. adiacens*; Ge, *G. elegans*; Ad, *A. defectiva*; Sm, *S. mutans*; Sg, *S. gordonii*.

As evident from confocal microscopy and visual inspection of crystal violet-stained biofilm preparations (Figure 2), all five bacterial species formed biofilm after 3 days of incubation. *G. adiacens* and *A. defectiva* formed lowest and highest amounts of biofilm, respectively (Figure 2). Further, *A. defectiva* biofilm mass was significantly higher than that of all other species except *S. gordonii*. Interestingly, crystal violet staining results were different from Syto-9 staining. This discrepancy is probably because crystal violet mainly stains complete EPS in the biofilm matrix while Syto-9 is a nucleic acid stain. Further, when we tested the effect of BITC on biofilm formation of the species, the inhibitory effect was not evident on the biofilms (Figure 3). No significant difference was seen between the quantities of BITC-treated and -untreated biofilms.

### Effect of BITC Treatment on the Expression of Biofilm-Associated Genes

**Figure 4:**
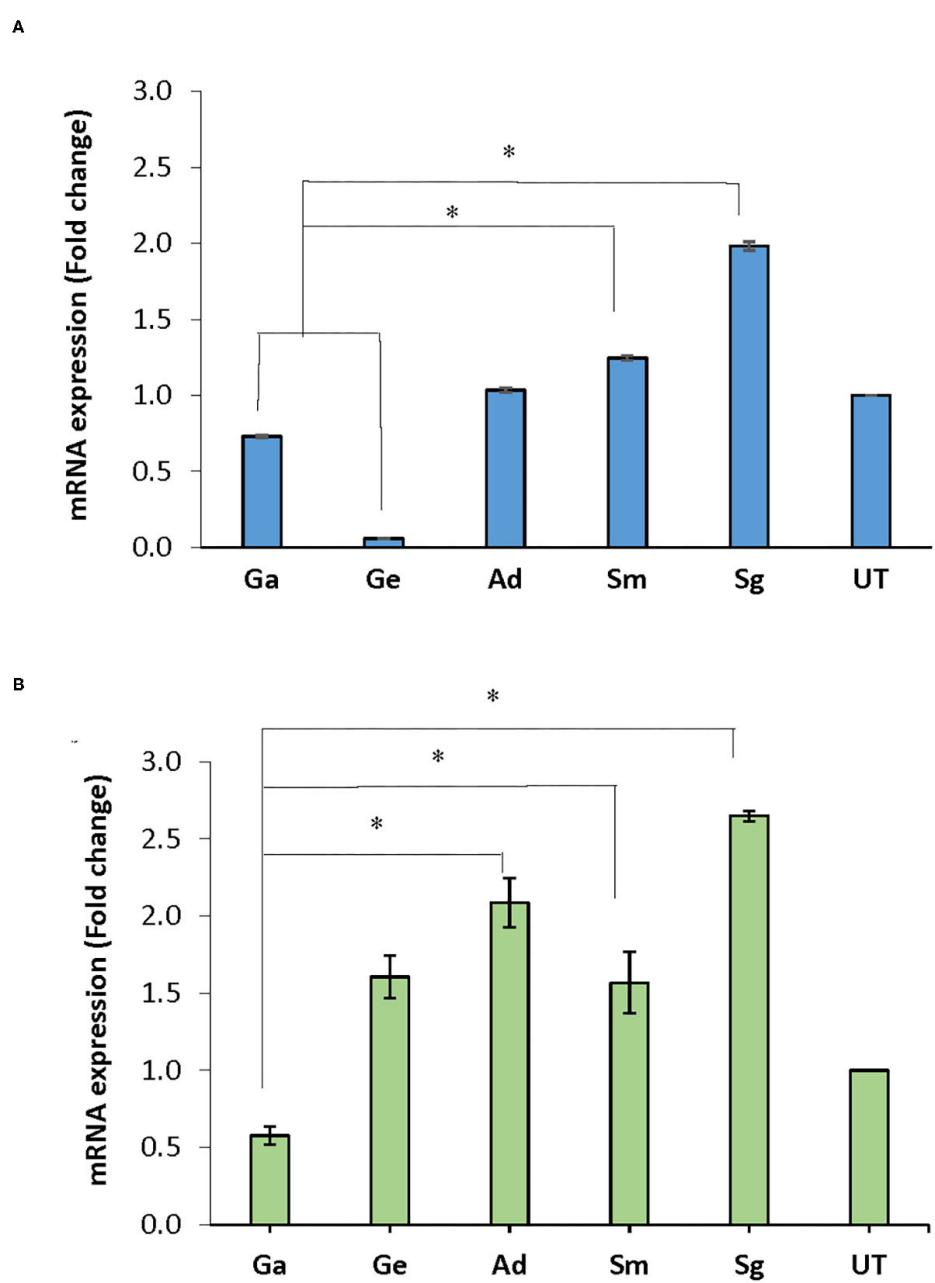
mRNA expression of *nox* and *fbp* genes. Mature biofilms of all strains were treated with BITC (10 μM) for 2 h in 5% CO_2_ at 37°C. The biofilms were scraped off from the wells, RNA *later*® was added to the biofilms and preserved at −80°C until used. RNA purified from the samples was converted to cDNA and expression of *nox* **(A)** and *fbp* **(B)** genes was assessed by Real-time PCR quantification. Ga, *G. adiacens*; Ge, *G. elegans*; Ad, *A. defectiva*; Sm, *S. mutans*; Sg, *S. gordonii*; UT, untreated. *P < 0.05.

Following treatment of the biofilms with BITC, *nox* mRNA expression in *A. defectiva, S. mutans*, and *S. gordonii* increased by 1.03, 1.25, 1.98 folds, respectively, compared to the untreated control after normalization with the housekeeping gene 16S rRNA. However, in *G. adiacens* and *G. elegans*, the expression decreased by 0.73 and 0.06-fold, respectively (Figure 4A). The *nox* mRNA expression in *S. gordonii* and *S. mutans* was significantly higher than in *G. adiacens* and *G. elegans* (*P* < 0.05). The expression of *fbp* mRNA was 1.61, 1.57, 2.09, and 2.65-folds higher in *G. elegans, S. mutans, A. defectiva*, and *S. gordonii*, respectively, when compared to the untreated control.

Similar to *nox, fbp* expression decreased 0.58-folds in *G. adiacens* (Figure 4B). The *fbp* mRNA levels in *A. defectiva, S. mutans*, and *S. gordonii* were significantly (*P* < 0.05) higher than in *G. adiacens*.

## Discussion

Dental plaque biofilms are the important etiologic factor in oral infectious diseases. Bacteria embedded in multilayered biofilms are difficult to kill because of the inherent resistance of the biofilm matrix toward antimicrobials. The present study demonstrated that the biofilms of three of the five bacterial species tested showed an increase in expression of the *nox* gene, which encodes for NADH oxidase and four bacterial species showed an increase of the *fbp* gene, encoding fibronectin binding protein following treatment with BITC. Altered gene expression due to antbiotic treatment of biofilms has been reported previously [11, 21, 22]. Further, like antibiotics, antimicrobials are also known to modify the expression of biofilm and virulence-associated genes in bacteria [12, 13, 23]. The natural antimicrobial BITC has been shown to exert potential health benefits on humans [22, 24] in addition to its antimicrobial activity [25, 26]. Recently, the expression differences of pathogenicity-related genes in *Salmonella typhimurium* upon BITC treatment was explored using transcriptome analysis and phenotypic validation like motility and biofilm formation characteristics. Differentially expressed genes including down- and upregulated were noticed in BITC treated group in comparison to the control group [27]. Similarly, downregulated expression of virulence factors was also observed in BITC treated *Staphylococcus aureus* experimental group [28].

We subjected all strains to the same concentration (10 μM) of BITC, which was chosen based on previous studies [16]. Even though our screening experiments showed that the inhibitory zones ranged 10–15 mm, no significant reduction in biofilm mass was seen at this concentration of BITC (Figure 3). Thus, we were prompted to study the expression of the biofilm-associated genes *nox* and *fbp* since the biofilm growth was not inhibited. Importantly, this eliminates the possibility that the reduced gene expression observed in some species in this study may be due to cell death.

We chose NADH oxidase (*nox*) and fibronectin (*fbp*) since their role in biofilm formation and virulence has been known in other bacterial species [14, 15]. In mutants lacking *nox* and *fbp*, biofilm formation was significantly decreased, which appeared to be due to reduced expression of a certain biofilm-associated genes [14]. In our study, when biofilms were treated with BITC, the overall structure of the biofilms was intact and no signs of biofilm detachment were seen upon visual inspection by stereo microscopy. While the expression of *nox* genes increased in *S. gordonii, S. mutans* and *A. defectiva*, a decreased expression was found in *G. adiacens* and *G. elegans* compared to the untreated control. In case of *fbp* genes, the increased expression was noticed in *S. gordonii, S. mutans, A. defective and G. elegans* while the expression was found to be decreased in *G. adiacens* alone compared to the untreated control. In the human oral cavity, bacteria need to cope with oxidative stress caused by diatomic oxygen as well as large amounts of H_2_O_2_ produced by competitor species. NADH oxidase is an important oxygen-metabolizing enzyme that plays a critical role in reducing dissolved oxygen in dental plaque [29]. Further, *nox* is also involved in the regulation of competence in some streptococci [30]. The increased expression of nox genes was reported in biofilm cells and under aerated and static aerobic conditions when compared to planktonic cells and anaerobic conditions [19]. Thus, our results suggest that *S. gordonii, S. mutans* and *A. defectiva* and *G. elegans* in which the gene expressions were increased, are possibly better equipped against antimicrobial assault. Increased expression of *nox* may indicate that these species produce higher amounts of NADH oxidase to combat an increased oxygen stress upon treatment with antimicrobials [31, 32]. Similarly, antibiotic treatment is also known to cause an increased expression of *fbp* genes in some bacterial species, enhancing adhesion efficacy of the bacterial cells [33, 34]. On the contrary, *G. elegans* and *G. adiacens*, which showed reduced expression of *nox* and *G. adiacens* of *fbp* upon BITC treatment, possibly are less capable of tailoring their gene expression upon treatment with antimicrobials.

A major limitation of this study is the lack of overall effect of BITC treatment on gene expression by microarray or transcriptome analysis, which may throw light on more interesting genes. Nevertheless, the two genes we studied, *nox* and *fbp* are known to be important for biofilm life in other species. In our ongoing study, we are aiming to show causality between upregulation of the expression of these two and several other genes upon antimicrobial treatment by constructing specific deletion mutants.

## Conclusion

Biofilms of the studied oral bacteria were not significantly killed in the presence of BITC.

Increased expression of the biofilm-associated genes *nox* and *fbp* following BITC treatment suggests a possible biological significance of the genes in the biofilm life style of these species. Further investigation is needed to understand the role of *nox* and *fbp* genes when oral bacterial biofilms are exposed to BITC. Further, molecular mechanisms of species-specific regulatory effects of BITC treatment on the expression of the biofilm-associated genes may offer a greater insight.

## Data Availability Statement

The raw data used in preparing this article will be made available by the authors, without undue reservation.

## Author Contributions

HA: investigation, formal analysis, and original draft. EA: investigation, formal analysis, and original draft-review and editing. RB: methodology, data curation, and formal analysis. AA: project administration, resources, and original draft-review and editing. MK: conceptualization, funding acquisition, supervision, and original draft-review and editing. All authors contributed to the article and approved the submitted version.

## Funding

This study was funded by the Kuwait University Grant SRUL 01/14.

## Conflict of Interest

The authors declare that the research was conducted in the absence of any commercial or financial relationships that could be construed as a potential conflict of interest.

## Publisher's Note

All claims expressed in this article are solely those of the authors and do not necessarily represent those of their affiliated organizations, or those of the publisher, the editors and the reviewers. Any product that may be evaluated in this article, or claim that may be made by its manufacturer, is not guaranteed or endorsed by the publisher.
